# Combining constraint-induced movement therapy and action-observation training in children with unilateral cerebral palsy: a randomized controlled trial

**DOI:** 10.1186/s12887-018-1228-2

**Published:** 2018-07-31

**Authors:** Cristina Simon-Martinez, Lisa Mailleux, Els Ortibus, Anna Fehrenbach, Giuseppina Sgandurra, Giovanni Cioni, Kaat Desloovere, Nicole Wenderoth, Philippe Demaerel, Stefan Sunaert, Guy Molenaers, Hilde Feys, Katrijn Klingels

**Affiliations:** 1 Department of Rehabilitation Sciences, KU Leuven - University of Leuven, Leuven, Belgium; 2 Department of Development and Regeneration, KU Leuven - University of Leuven, Leuven, Belgium; 3 Department of Developmental Neuroscience, IRCCS Fondazione Stella Maris, Calambrone, Italy; 4 Department of Clinical and Experimental Medicine, University of Pisa, Pisa, Italy; 5 Clinical Motion Analysis Laboratory, University Hospitals Leuven, Pellenberg, Belgium; 6 Neural Control of Movement Lab, Department of Health Sciences and Technology, ETH, Zurich, Switzerland; 7 Department of Radiology, University Hospitals Leuven, Leuven, Belgium; 8 Rehabilitation Research Centre, BIOMED, Hasselt University, Diepenbeek, Belgium

**Keywords:** Unilateral cerebral palsy, Upper extremity, Neuroimaging, Intensive therapy, Brain injuries, Treatment outcome

## Abstract

**Background:**

Upper limb (UL) deficits in children with unilateral cerebral palsy (uCP) have traditionally been targeted with motor execution treatment models, such as modified Constraint-Induced Movement Therapy (mCIMT). However, new approaches based on a neurophysiological model such as Action-Observation Training (AOT) may provide new opportunities for enhanced motor learning. The aim of this study is to describe a randomised controlled trial (RCT) protocol investigating the effects of an intensive treatment model, combining mCIMT and AOT compared to mCIMT alone on UL function in children with uCP. Additionally, the role of neurological factors as potential biomarkers of treatment response will be analysed.

**Methods:**

An evaluator-blinded RCT will be conducted in 42 children aged between 6 and 12 years. Before randomization, children will be stratified according to their House Functional Classification Scale, age and type of corticospinal tract wiring. A 2-week day-camp will be set up in which children receive intensive mCIMT therapy for 6 hours a day on 9 out of 11 consecutive days (54 h) including AOT or control condition (15 h). During AOT, these children watch video sequences showing goal-directed actions and subsequently execute the observed actions with the more impaired UL. The control group performs the same actions after watching computer games without human motion. The primary outcome measure will be the Assisting Hand Assessment. Secondary outcomes comprise clinical assessments across body function, activity and participation level of the International Classification of Function, Disability and Health. Furthermore, to quantitatively evaluate UL movement patterns, a three-dimensional motion analysis will be conducted. UL function will be assessed at baseline, immediately before and after intervention and at 6 months follow up. Brain imaging comprising structural and functional connectivity measures as well as Transcranial Magnetic Stimulation (TMS) to evaluate corticospinal tract wiring will be acquired before the intervention.

**Discussion:**

This paper describes the methodology of an RCT with two main objectives: (1) to evaluate the added value of AOT to mCIMT on UL outcome in children with uCP and (2) to investigate the role of neurological factors as potential biomarkers of treatment response.

**Trial registration:**

NCT03256357 registered on 21st August 2017 (retrospectively registered).

**Electronic supplementary material:**

The online version of this article (10.1186/s12887-018-1228-2) contains supplementary material, which is available to authorized users.

## Background

Cerebral palsy (CP) is the most common physical disability in childhood, occurring in 1–3 per 1000 live births [1]. Unilateral CP (uCP) accounts for 38% of the cases [2]. These children present with motor and sensory impairments predominantly on one side of the body, which are usually more pronounced in the upper limb (UL) [3]. These sensorimotor impairments typically lead to limited capability to perform daily tasks, having an impact on their participation and quality of life [4]. Hence, over the last decade, research into UL interventions for children with uCP has grown exponentially. One of the most popular treatment modalities amongst clinicians and researchers is modified Constraint-Induced Movement Therapy (mCIMT) [5]. mCIMT constrains the less impaired hand and targets intensive unimanual task-related practice with the more impaired UL. Despite increasing evidence proving the effectiveness of mCIMT in children with uCP, variable treatment outcomes have been reported [5–7].

The main focus of mCIMT is motor execution, although it has been shown that children with uCP also present with deficits in motor representations involved in the planning of movements [8]. Treatment modalities targeting motor representations might therefore further enhance the learning and rehabilitation process. Based on neurophysiological findings, it has been suggested that the use of systematic observations of meaningful actions followed by their execution, i.e. action-observation training (AOT), may accelerate the process of motor learning [9, 10]. Brain areas responsible for this action observation–action execution matching system are known as the mirror neuron system and include a bilateral network within the frontal premotor, parietal and temporo-occipital cortex underlying action observation [11]. Three recent studies using AOT in children with uCP have shown promising results [12–14]. However, it remains unclear whether combining mCIMT with a treatment modality targeting motor representation, such as AOT, will augment the treatment effects and result in longer retention.

Several studies investigating the efficacy of mCIMT in children with uCP, have reported a large inter-individual variability in treatment response [6, 15–17]. These studies have suggested that children with poorer UL function at baseline may benefit more from mCIMT. Moreover, some studies have investigated whether neurological factors, e.g. corticospinal tract (CST) wiring pattern or brain lesion characteristics, may determine treatment response. Despite the increasing number of studies investigating this research question, results are still contradicting. Two studies hypothesized that in children with an ipsilateral wiring pattern, constraining the less impaired UL may drive down primary motor cortex activity controlling both ULs, and thus possibly preventing improvement of the more impaired UL [18, 19]. In contrast, Islam et al. reported improved UL function after mCIMT irrespective of the CST wiring patterns [20], highlighting the importance of considering other relevant neurological factors. Interestingly, a few studies have already investigated the potential role of structural and functional connectivity in predicting treatment response, reporting that especially children with more affected structural and functional connectivity improved after mCIMT [21–23]. Notwithstanding the valuable insights reported by these studies, their sample sizes were relatively small. Moreover, the combination of structural and functional connectivity with CST wiring pattern to predict treatment outcome in children with uCP has not yet been investigated.

Furthermore, UL function has thus far mostly been evaluated using clinical scales on body function and activity level according to the International Classification of Functioning, Disability and Health (ICF) model. Whilst these clinical scales have been proven valid and reliable, they lack the information on anatomical motions at the single joint level. Moreover, they do not capture the complexity of UL motion, involving the coordinated interaction of movement sequences of multiple degrees of freedom. Hence, a more quantitative assessment, such as three-dimensional movement analysis (3DMA), may provide a better understanding of the changes that occur at the joint level and thus contribute to further insights on the effectiveness of UL treatment programs in children with uCP.

This study protocol describes the set-up for a single blind randomized controlled trial (RCT) comparing the effects of mCIMT with or without AOT on UL function using both clinical and kinematic outcomes. The first objective is to examine whether combining mCIMT with AOT will augment the treatment effects and result in longer retention. Secondly, the potential role of the anatomical characterization of the brain lesion, structural and functional connectivity and the CST wiring in predicting treatment response will be investigated. These findings might aid in guiding patient selection for tailor-made intervention programs in children with uCP.

### Hypothesis

The RCT will address the following research hypotheses:

1. mCIMT, in combination with AOT, augments the treatment effects immediately after intervention and results in improved UL function with longer retention beyond mCIMT alone.
2. The combination of neurological predictors, i.e. neuroanatomical brain lesion characteristics, structural and functional connectivity and CST wiring pattern, better determines treatment response compared to clinical predictors.
3. Children with a bilateral and ipsilateral CST wiring respond less to the treatment compared to children with contralateral CST wiring in both intervention groups.
4. In children with more lesions or disturbed connectivity in the areas involving the mirror neuron system, AOT is not as effective as in those with less lesions in this area.

## Methods

### Study design

**Fig 1:**
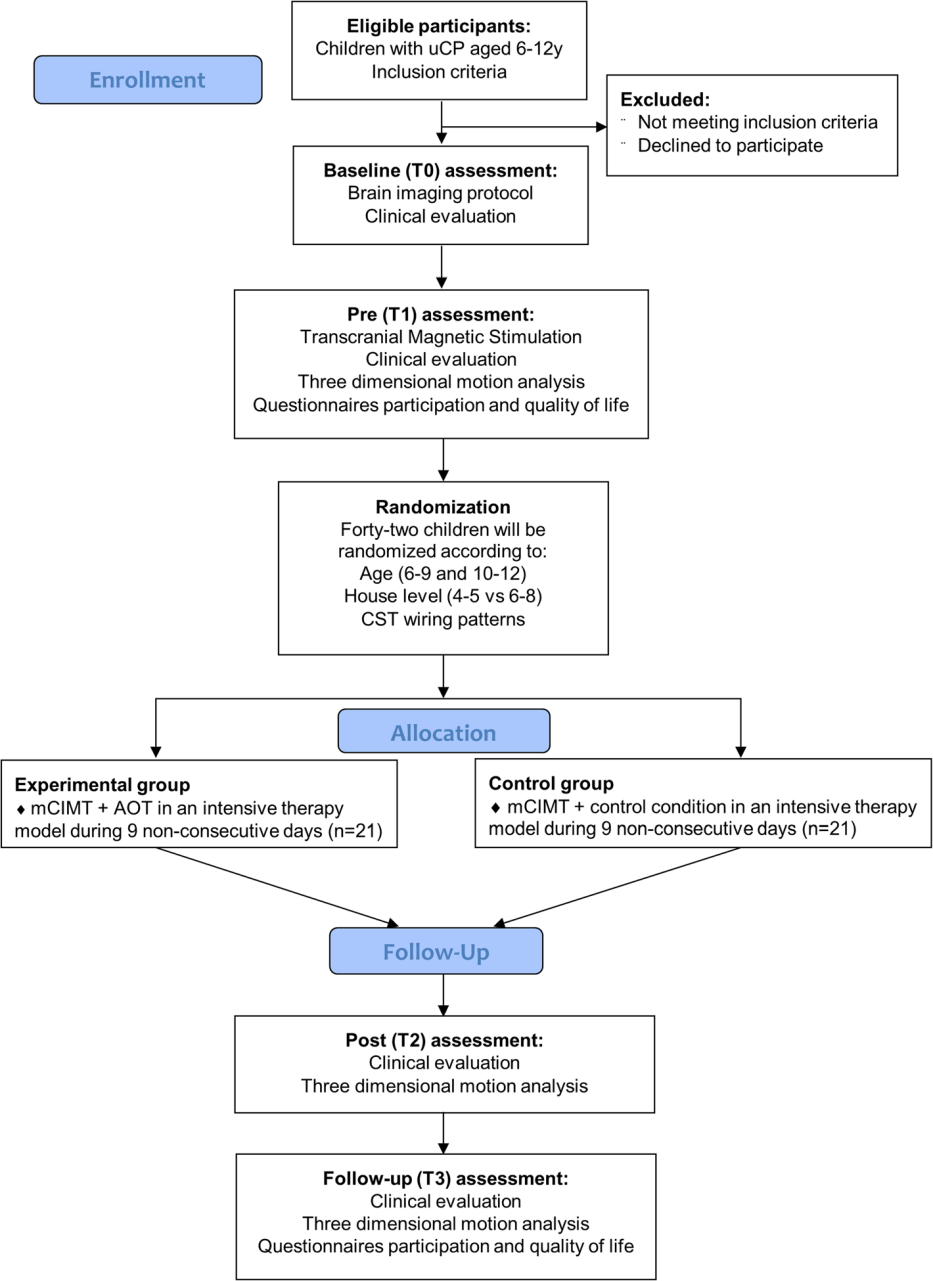
Flow-chart of the described RCT following the CONSORT guidelines. Abbreviations: uCP, unilateral cerebral palsy; CST, corticospinal tract; mCIMT, modified constraint-induced movement therapy; AOT, Action-Observation Training

An evaluator-blinded RCT will be implemented comparing mCIMT with and without AOT on UL function in children with uCP. Ethical approval was obtained by the Ethical Committee of the University Hospitals Leuven (S56513). Before entering the study, written informed consent from all parents or care givers and verbal assent from all the participants will be obtained. Assessments will be performed at T0 (baseline, 3–4 month before the intervention onset), T1 (within 4 days before the intervention), T2 (within 4 days after the intervention) and T3 (6 months after the intervention). A summary of the experimental design is described in Fig. 1 and an overview of the outcome measures are presented in Table 1.

**Table 1:** Overview of the assessments at each time-point

		Baseline (T0)	Pre-evaluation (T1)	Post-evaluation (T2)	Follow-up evaluation (T3)
Descriptive characteristics			MACS HFC CVI Sensory assessment Mirror Movements		
Outcome measures	Body Function and Structure	pROM, muscle strength, grip force and spasticity	pROM, muscle strength, grip force and spasticity	pROM, muscle strength, grip force and spasticity	pROM, muscle strength, grip force and spasticity
	Activity	AHA, MA2, JTHFT, ABILHAND-Kids, CHEQ	AHA, MA2, JTHFT, ABILHAND-Kids, CHEQ and Tyneside Pegboards	AHA, MA2, JTHFT, ABILHAND-Kids, CHEQ and Tyneside Pegboards	AHA, MA2, JTHFT, ABILHAND-Kids, CHEQ and Tyneside Pegboards
	Participation		CPQOL and Life-H		CPQOL and Life-H
	Motion analysis		3DMA	3DMA	3DMA
Neurological predictors		sMRI, dMRI and rsfMRI	TMS		

*Abbreviations*: *MACS* Manual Ability Classification System, *HFC* House Functional Classification, *CVI* Cerebral Visual Impairment, *pROM* passive range of motion, *AHA* Assisting Hand Assessment, *MA2* Melbourne Assessment 2, *JTHFT* Jebsen-Taylor Hand Function Test, *CHEQ* Child Hand-use Experience Questionnaire, *CPQOL* CP quality of life questionnaire, *Life-H* Life Habits questionnaire, *3DMA* three-dimensional motion analysis, *sMRI* structural MRI, *dMRI* diffusion MRI, *rsfMRI* resting-state functional MRI, *TMS* transcranial magnetic stimulation

### Study sample and recruitment

Children with spastic uCP will be recruited via the CP-care program of the University Hospitals Leuven. They will be selected upon the following inclusion criteria: (1) confirmed diagnosis of uCP; (2) aged 6–12 years at time of baseline assessment; (3) sufficient cooperation to comprehend and complete the test procedure and cooperate in the camp activities; (4) minimal ability to actively grasp and stabilize an object with the more impaired hand (House Functional Classification Score ≥ 4). Children will be excluded in case of previous UL surgery in the last 2 years, or botulinum toxin-A injections 6 months prior to the baseline assessment.

### Randomisation

Children will be assigned using stratified random sampling. Before intervention (T1), children will be first stratified according to the House Functional Classification Scale (4–5 vs. 6–7), age (6-9y vs. 10–12 y), and the type of CST wiring pattern (contralateral, bilateral and ipsilateral) assessed by Transcranial Magnetic Stimulation (TMS) to maximize homogeneity and minimize group differences at baseline. A permuted block design of two will then be used, created by a computer random number generator to randomize the participants to the mCIMT+AOT or mCIMT alone group within each stratum. Randomization will be performed by an independent person who is not involved in the selection procedure and cannot access the clinical information of the children.

### Sample size

Sample size estimate is based on the primary endpoint, which is defined as the immediate effect of the intervention on the primary outcome measure, i.e. bimanual performance measured with the Assisting Hand Assessment (AHA). The smallest detectable difference has been reported to be 5 AHA units [24]. A previous intervention study of intensive therapy in children with uCP [6], reported a standard deviation of 5.5 AHA units, which would translate into an effect size of 0.9. With this effect size, an alpha-level of 0.05, and a statistical power of 0.80, a sample size of 21 children is needed in each group to detect a difference equal to or larger than the smallest detectable difference of 5 AHA units between groups [24, 25]. Sample size estimates were calculated with G*Power [26, 27].

### Blinding

In order to blind parents and children to group allocation, they will only be informed about the general description of the study design. However, they will not be informed about the type of observation the children eventually receive (AOT or control condition). All therapists and study personnel assisting during the intervention will not be blinded of group allocation. One blinded, experienced physiotherapist, not involved in the camp activities, will assess UL function at the four different time points. Video-based clinical scales (AHA and Melbourne Assessment 2) will be scored afterwards by another evaluator, blinded to group allocation and time point of the assessment. The 3DMA will be performed by two experienced physiotherapists not blinded to group allocation, as these analyses are fully automated.

### Treatment protocol

A day camp model will be used during which children receive intensive therapy for 6 hours a day, for 9 out of 11 consecutive days, with no therapy during the weekend (total of 54 h of therapy). Child/therapist ratio will be 1:1 to secure individual guidance. Experienced paediatric physiotherapists will lead the camps, assisted by physiotherapy master students, specialized in paediatric rehabilitation.

During the camps, all children wear a tailor-made hand splint on the less impaired UL while performing unimanual exercises based on (1) shaping and repetitive practice during individual therapy (9 h), (2) group activities (30 h) and (3) action-observation training or control condition (15 h). The theme throughout the camp is ‘Zora’, a rehabilitation robot that will welcome and motivate the children to engage in the activities. The splint is a rigid orthosis, individually adjusted and covering fingers, thumb, and wrist.

#### Individual therapy

One hour per day the child receives individual therapy based on motor learning principles of shaping and repetitive practice. Four goals will be trained that focus on the most commonly reported UL problems: active wrist and elbow extension, forearm supination, grip strength and fine motor tasks. The main investigators developed a manual encompassing exercises for these four goals embedded in functional activities. Individual guidelines for each child will be set up, based on baseline body function measures and video-based assessments of the Melbourne Assessment 2 (see evaluation for more details). Each child will exercise the four goals within a rotation system, in which each goal is practiced for 15 min taking the individual guidelines of the child into account. The degree of difficulty of the exercises and therapy equipment will be adapted daily to the child’s progress.

#### Group activities

The group activities will consist of varied activities such as painting, cooking, crafts and outdoor games. These activities will be selected and adapted to stimulate intensive use of the more impaired hand. This was further ensured by the one-on-one guidance. The activities will be uniquely performed with the more impaired hand. In activities demanding the use of two hands, the children will cooperate in pairs with each other or with the therapist.

#### Action-observation training

Children in the experimental group will receive a total of 15 AOT sessions of one hour, which is 1 or 2 h per camp day. The AOT program will be in line with the one described by Sgandurra et al. [13, 28]. However, the bimanual tasks will be replaced by unimanual activities, in order to keep the focus on unimanual training. During AOT, the children will watch video sequences showing unimanual goal directed actions. Two series of activity sets are developed, adapted according to the UL functional level of the child: one for children with House Functional Classification 4 or 5 (see Additional file 1: Table S1) and one for those with House Functional Classification 6 to 8 (see Additional file 2: Table S2). The set-up and the goal of the activities is similar, although the type of movement is simplified for the children classified in level 4–5. Both in the videos as well as during the execution of the tasks, all the material is placed on dark surface to highlight the contrast and facilitate the focus on the activity, in particular for those with a visual and/or attention problem. To avoid potential mental rotation, all videos will be shown in the perspective of the child (i.e. first-person perspective and side of the impaired hand is performing the action, where only the arm and hand are visible). The children will sit 50 cm in front of a computer screen of 22 in.. A therapist will sit next to the child on the more impaired side. In total, the AOT will consist of 15 tasks, one for each session, and each task will consist of three sub-activities. One action will be repeated for a total duration of 3 minutes. After watching this video sequence, the child will execute the observed actions with the more impaired UL repeatedly for 3 minutes. Each video will be performed twice. As such, a total of six video sequences are shown during one therapy session. While watching the videos, the therapist will keep the attention of the child focused on the shown actions. During the execution of the action, the therapist will verbally stimulate the child without giving any suggestive remarks (regarding movement quality) or providing a demonstration.

The children in the control group will watch video games not showing any human movements and not requiring any manual actions of the child because the therapist seated next to the child will control the keyboard and mouse. Afterwards, these children will practice the same tailored actions for 3 minutes in the same order as the experimental group. Verbal instructions will be given by the therapist without suggestive remarks or a demonstration of the task performance.

### Clinical evaluation

The clinical evaluation takes place in the Clinical Motion Analysis Laboratory of the University Hospitals Leuven.

#### Descriptive and clinical characteristics

General patient’s characteristics, such as age, more impaired side, and co-morbidities, will be recorded at baseline. Children will be classified according to the House Functional Classification System (HFC) and the Manual Ability Classification System (MACS). The HFC is a nine-level functional classification system, describing the role of the assessed hand as a passive or active assist in bimanual activities from 0 ‘does not use’ to 8 ‘uses hand completely independently without reference to the other hand’. This scale has been found to be reliable to classify unimanual function in children with spastic CP [29, 30]. The MACS reliably classifies the ability to handle objects in daily activities in children with CP between 4 and 18 years [31, 32]. It ranks the children on a five-level scale (level I = ‘Handles objects easily and successfully’; level V = ‘Does not handle objects and has severely limited ability to perform even simple actions’).

#### Cerebral visual impairment questionnaire

The Cerebral Visual Impairment (CVI) questionnaire was developed to screen children who may suffer from this impairment. This questionnaire is filled in by the parents and consists of 46 closed ended items clustered in six domains, evaluating visual attitude, ventral and dorsal stream functions, complex visuomotor abilities, use of other senses, and associated CVI characteristics. The CVI questionnaire has shown good sensitivity and specificity [33]. This questionnaire will serve as a starting point to determine whether the child may present with CVI and, therefore, may have some difficulties in observing the videos of the action-observation training.

#### Sensory function

Sensory assessments will be measured before the intervention (T1). They will comprise exteroception (tactile sense), proprioception (movement sense), two-point discrimination (Aesthesiometer®) and stereognosis (tactile object identification). These sensory assessments will be carried out following the protocol defined by Klingels et al. [34], which has been shown to be reliable in this population. Furthermore, a kit of 20 nylon monofilaments (0.04 g - 300 g) (Jamar® Monofilaments, Sammons Preston, Rolyan, Bolingbrook, IL, USA) will be used to determine threshold values for touch sensation [35]. This assessment has also shown to be reliable in children with uCP [36].

#### Mirror movements

Mirror movements will be evaluated before the intervention (T1). First, the occurrence of mirror movements will be scored during three unimanual tasks: (1) fist opening and clenching, (2) thumb-finger opposition, and (3) alternate finger tapping on a table surface. Each task will be performed five times with both hands separately, starting with the more impaired hand. Task execution will be video recorded and mirror movements will be scored following the 4-point ordinal scale of Woods and Teuber [37]. Second, the Grip Force Tracking Device (GriFT Device) will be used to evaluate mirror movements during repetitive unimanual squeezing while playing a computer game [38]. This portable device consists of two identical handles containing force sensors. First, the maximum voluntary contraction of each hand is calculated. Next, the children are asked to repetitively squeeze with one hand while playing a computer game. The rhythm is determined by a visual cue with a frequency of 0.67 Hz at 15% of the previously determined maximum voluntary contraction. Mirror movement characteristics such as frequency, strength and temporal features (synchronization and time lag) will be extracted, following the protocol described by Jaspers et al. [38].

### Outcome measures

UL function will be comprehensively evaluated on the levels of body function and structure, activity and participation following the ICF model.

#### Primary outcome measure

The AHA will be the primary outcome measure and it will be evaluated at every time point. The AHA assesses how effectively the more impaired hand is used in bimanual activities [25, 39, 40]. The spontaneous use is evaluated during a semi-structured play session with standardized toys requiring bimanual handling. The performance is video recorded and scored afterwards. Given the age range of the participants of this study, the School Kids AHA will be used, scored with version 5.0. This version includes 20 items that are scored form 0 (‘does not do’) to 4 (‘effective use’), and it has been shown to be valid and reliable [25, 40].

#### Secondary outcome measures

##### UL assessment at body function and structure level

UL motor impairments will be assessed at every time point and include (1) passive range of motion (pROM), (2) muscle tone, (3) muscle strength and (4) grip strength. All assessments will be executed following a valid and reliable protocol in children with uCP defined by Klingels et al. [34]. A universal goniometer will be used to evaluate pROM of the shoulder (flexion, abduction, internal and external rotation) elbow (flexion and extension), forearm (pronation and supination) and wrist (flexion and extension). Muscle tone will be assessed using the Modified Ashworth Scale [41] for muscle groups of the shoulder (extensors, adductors, abductors, external and internal rotators), elbow (flexors, extensors and pronators), wrist (flexors and extensors) and hand (finger flexors and thumb adductors). Muscle strength will be evaluated using manual muscle testing [42] according to the 8-point ordinal scale of the Medical Research Council. Muscle groups of the shoulder (flexors, adductors and abductors), elbow (flexors, extensors, supinators and pronators) and wrist (flexors and extensors) will be assessed. Finally, maximum grip strength will be assessed using the Jamar® hydraulic hand dynamometer (Sammons Preston, Rolyan, Bolingbrook, IL, USA). The mean of three maximum contractions will be calculated for both hands. Furthermore, to calculate the Static Fatigue Index as described by Severijns et al. [43], a 30 s sustained contraction will be performed with a digital hand grip module (E-link, Biometrics Ltd., Newport, UK). The sustained contraction will be evaluated at time points T1, T2 and T3.

##### UL assessment at activity level

UL activity assessments will include measures of unimanual capacity, bimanual performance and manual ability.

- Melbourne Assessment 2

The Melbourne Assessment 2 (MA2) is a criterion-referenced test designed for children with uCP aged 2.5 to 15 years [44]. This scale measures unimanual capacity and has been proven valid and reliable for this population [45]. The MA2 assesses UL movement quality by means of 14 unimanual tasks, including 30 movement scores grouped across four subscales: range of motion, accuracy, dexterity and fluency. Each sub-score is converted into a percentage. The performance is video recorded and subsequently scored. The MA2 will be measured at every time point.

- Jebsen-Taylor hand function test

The Jebsen-Taylor hand function test (JTHFT) measures movement speed during six unimanual tasks [46, 47]. As similar to other studies, a modified version for children with uCP will be used. In the modified version, the writing task is removed, and the time to carry out each teak is reduced from 3 to 2 min to avoid frustration [16, 48]. This test uses standardized material and time needed to perform the task is directly recorded. Practice trials are not allowed. The JTHFT has established construct, content validity and reliability [16]. This test will be evaluated at every time point.

- Tyneside pegboard test

The Tyneside pegboard test will be used to quantify unimanual and bimanual dexterity. The Tyneside pegboard test is an adapted 9-hole pegboard test, where two adjacent boards are placed next to each other. In the unimanual task, the child moves the pegs from one board to the other using first the less impaired and then the more impaired hand, recorded separately. The unimanual task will be repeated three times with different peg sizes (large, medium and small). For the asymmetric bimanual task, the large pegs will be picked up from one board, passed through a hole in a Perspex® divider placed between the boards, and inserted into the second board with the other hand. Children will be instructed to perform the tasks as fast as possible without paying attention to the order of lifting and inserting the pegs. The test is electronically timed and results are outputted using a custom-written software (Institute of Neuroscience, Newcastle University, Newcastle upon Tyne, United Kingdom) [49]. This test will be evaluated at T1, T2 and T3.

- ABILHAND-Kids Questionnaire

The ABILHAND-Kids questionnaire is developed to assess manual ability in children with CP aged 6 to 15 years. It comprises 21 mainly bimanual daily activities. The difficulty experienced by the child to perform the required tasks is rated on a 3-point ordinal scale by the parents [50]. A Rash model was used to validate the ABILHAND-Kids questionnaire and its reliability and reproducibility over time has been shown [50]. This questionnaire will be evaluated at every time point.

- Children’s Hand-use Experience Questionnaire

The Children’s Hand-use Experience Questionnaire (CHEQ) is an online questionnaire that captures the child’s experience of using the more impaired hand during bimanual activities (available online at http://www.cheq.se). Parents will answer 29 questions to describe how independently the activities are performed. Each question has three sub-questions, on a 4-point rating scale, measuring (i) hand use, (ii) time use in comparison to peers and (iii) experience of feeling bothered when doing the activity. A Rash model was used to validate the CHEQ and its reliability has been shown [51]. This questionnaire will be evaluated at every time point.

##### Three-dimensional motion analysis

Upper Limb Three-Dimensional Motion Analysis (UL-3DMA) will be conducted at T1, T2 and T3. A custom-made chair with foot and back-support is used to perform the measurements in a standardized sitting position. A total of 17 reflective markers (14 mm diameter) are attached to the trunk, acromion, upper arm, forearm, and hand. Next, several static calibration trials are conducted to identify anatomical landmarks of interest, following the guidelines of the International Society of Biomechanics [52]. The movement protocol contains eight tasks: three reaching tasks (forwards, RF; upwards, RU; sideways, RS), two reach-to-grasp tasks (grasp a sphere, RGS; grasp a vertical cylinder, RGV) and three daily-life activities mimicking tasks (hand-to-head, HTH; hand-to-mouth, HTM; hand-to-shoulder, HTS). Each task was performed four times within two trials, resulting in eight movement repetitions per task. Tasks were executed with the impaired UL at self-selected speed. Each task is started in upright sitting with 90° of hip and knee flexion, with the impaired hand on the ipsilateral knee. This protocol has been proven reliable in children with uCP [53]. To record motion, 12 to 15 Vicon infrared cameras (Oxford Metrics, Oxford, UK) sampling at 100 Hz will be used to capture the UL movement patterns. Offline data processing will be performed with Vicon Nexus software (version 1.8.5, Oxford Metrics, Oxford, UK) and consists of a Woltring filtering routine with a predicted mean squared error of 10 mm^2^ [54], gap filling, and selection of the movement cycles (start (hand on ipsilateral knee) and end of each movement cycle). Task end-point is defined as follows: (1) touching the spherical object with the palm of the hand (RF, RU and RS), (2) grasping (sphere (RGS) or vertical cylinder (RGV)), and (3) touching different parts of the body (top of the head (HTH), mouth (HTM) or contralateral shoulder (HTS)). To avoid start and stop strategies of the child, only the middle two repetitions of each trial will be analysed, resulting in four analysed movement repetitions per task. Lastly, we time-normalize the movement cycles (0–100%) and calculate the root mean squared error (RMSE) of the kinematic angles of each cycle to compare it to the mean of the remaining 3 cycles (per task). As such, we retain the 3 cycles with the lowest RMSE for further analysis, which represent the most reliable movement patterns. The open source software ULEMA v1.1.9 [53, 55, 56] will be used to calculate the kinematics of five joints with a total of 13 angles: trunk (rotation, lateral flexion and flexion-extension), scapula (tilting, pro-retraction and rotation), shoulder (rotation, elevation plane and elevation), elbow (flexion-extension and pro-supination) and wrist (flexion-extension and ulnar-radial deviation). Spatiotemporal parameters, joint kinematics and summary indices will be calculated and used for statistical analysis.

##### Assessment of participation and quality of life

Participation and quality of life of the children will be evaluated at time points T1 (before) and T3 (follow-up).

- Participation

To evaluate changes in participation, parents will be asked to fill in the short version of the Life Habits (Life-H) questionnaire. This short version contains 64 items on life habits such as nutrition, fitness, personal care, mobility and community life. It uses a scoring system ranging from 0 (total impairment) to 9 (optimal participation) [57]. The Life-H has a good validity and a good internal consistency and a moderate test-retest reliability [4].

- Quality of life

To evaluate changes in quality of life, parents will be asked to fill in the Cerebral Palsy Quality of Life Questionnaire (CPQOL). The CPQOL is a condition-specific measure, designed for children with CP, that evaluates the well-being of children across seven areas of a child’s life: social well-being and acceptance, functioning, participation and physical health, emotional well-being, access to services, pain and impact of disability and family health [58]. In this study, the primary caregiver-proxy report, which contains 66 items, version for children aged 4–12 years will be used. The CPQOL has a high internal consistency and good test-retest reliability [58].

### Neurological predictors

A 3.0-T system (Achieva, Philips Medical Systems, Best, The Netherlands) will be used for image acquisition. The medical imaging protocol will include (1) structural magnetic resonance imaging (sMRI) for anatomical characterization (i.e. lesion timing, location and extent), (2) diffusion weighted imaging (dMRI) to evaluate white matter structural connectivity and (3) resting-state functional MRI (rsfMRI) analysing functional connectivity. To familiarize the children with the scanner situation, they will follow a training session prior to the scan, which consists of performing scan-related tasks similar to the protocol described by Theys et al. [59].

#### Structural MRI

Structural images will be acquired using three-dimensional fluid-attenuated inversion recovery (3D FLAIR) with following parameters: 321 sagittal slices, slice thickness = 1.2 mm, slice gap = 0.6 mm, repetition time = 4800 ms, echo time = 353 ms, field of view = 250 × 250 mm^2^, 1.1 × 1.1 × 0.56 mm^3^ voxel size, acquisition time = 5 min. In addition, magnetization prepared rapid gradient echo (MPRAGE) will be acquired with following parameters: 182 slices, slice thickness = 1.2 mm, slice gap = 0 mm, TR = 9.7 ms, TE = 4.6 ms, FOV: 250 × 250mm^2^, 0.98 × 0.98 × 1.2 voxel size, acquisition time = 6 min. Also, T2-weighted images will be obtained with following parameters: slice thickness 4 mm, TR = 6653 ms, TE = 100 ms, FOV = 250 × 250 mm2, 0.94 × 0.94 × 1.0 voxel size, acquisition time = 3 min.

Brain lesions will be first classified according to the timing of the lesion and the predominant pattern of damage as described by Krägeloh-Mann and Horber (2007) [60]: cortical malformations (first and second trimester of pregnancy), periventricular white matter (PWM) lesions (from late second till early third trimester) and cortical and deep grey matter (CDGM) lesions (around term age) and acquired brain lesions (between 28 days 3 years postnatally). Second, a more detailed evaluation of the brain lesion (i.e. location and extent) will be performed by a paediatric neurologist (EO) using the semi-quantitative MRI (sqMRI) scale developed by Fiori et al. (2014) [61]. The sqMRI scale consists of a graphical black and white template, adapted from the CH2 atlas [62] and a simple scoring system. In a first step, the lesion will be drawn onto the template, which consists of six axial slices. The boundaries of three layers (periventricular white matter, middle white matter and cortico-subcortical layer) and four lobes (frontal, parietal, temporal and occipital lobes) are marked on this template. Subsequently, for both hemispheres each layer in each lobe will be scored, resulting in a lobar score (range 0–3) and summed up to obtain a hemispheric score (range 0–12). The presence or absence of abnormalities of the lenticular and caudate nucleus, thalamus, posterior limb of internal capsule (PLIC) and brainstem, will be scored directly from the MRI scan as affected (score 1) or not affected (score 0), respectively (subcortical score, range 0–5). Also, the corpus callosum (anterior, middle and posterior section, range 0–3) and cerebellum (vermis, right and left hemisphere, range 0–3) will be evaluated directly from the MRI scan. Next, a total score for the affected and less affected hemisphere (range 0–17) can be calculated as the sum of the hemispheric and subcortical score of each respective hemisphere. Finally, the sum of all scores will result in the global score (range 0–40). Reliability and validity of the scale has already been established in children with uCP [61, 63, 64].

#### Diffusion weighted imaging

Diffusion weighted images (dMRI) will be acquired using a single shot spin echo sequence with the following parameters: slice thickness = 2.5 mm, TR = 8700 ms, TE = 116 ms, number of diffusion directions = 150, number of sagittal slices = 58, voxel size = 2.5 × 2.5 × 2.5 mm^3^, acquisition time = 18 min. Implemented b values are 700, 1000, and 2800 s/mm^2^, applied in 25, 40, and 75 uniformly distributed directions, respectively. In addition, 11 non-diffusion weighted images will be obtained. dMRI data will be pre-processed and analysed in ExploreDTI toolbox, version 4.8.6 (available for download at http://www.exploredti.com/download.htm). Diffusion metrics, such as fractional anisotropy and mean diffusivity of white matter tracts of interest (i.e. corpus callosum, corticospinal tract, medial lemniscus superior, thalamic radiations) will be calculated for both hemispheres using manually drawn regions of interest.

#### Resting state functional MRI

Resting-state function MRI (rsfMRI) images will be acquired using a T2*-weighted gradient-echo planar imaging sequence with the following parameters: TR = 1700 ms; TE = 30 ms; matrix size = 64 × 64; FOV = 230 mm; flip angle = 90°; slice thickness = 4 mm; no gap; axial slices = 30; number of functional volumes = 250; acquisition time = 7 min. Participants will be instructed to stay at rest, with eyes open, not to fall asleep and to think of nothing in particular. rsfMRI will be pre-processed with Statistical Parametric Mapping version 12 (SPM12) software [65]. Functional connectivity analysis will be computed with the CONN toolbox v17b [66, 67]. Correlation coefficients (indicating high versus low functional connectivity) will be determined among cortical and subcortical regions of interest within the sensorimotor network in the affected and less-affected hemisphere, which are relevant for UL function. Furthermore, the cortical areas involving the mirror neuron system will also be explored.

#### Transcranial magnetic stimulation

Single-pulse Transcranial Magnetic Stimulation (TMS) will be conducted to assess the CST wiring pattern. This assessment will be conducted only in the children with uCP who were eligible for this test, i.e. no implants in the body (metals, pacemaker, ventriculoperitoneal shunt) and no seizures within the last 2 years [68]. TMS will be performed using a MagStim 200 Stimulator (Magstim Ltd., Whitland, Wales, UK) equipped with a focal 70 mm figure-eight coil and a Bagnoli electromyography (EMG) system with two single differential surface electrodes (Delsys Inc., Natick, MA, USA). A Micro1401–3 acquisition unit and Spike software version 4.11 (Cambridge Electronic Design Limited, Cambridge, UK) were used to synchronize the TMS stimuli and the EMG data acquisition. Motor Evoked Potentials (MEPs) will be bilaterally recorded, using single differential surface EMG electrodes attached on the muscles adductor pollicis brevis of both hands.

We will follow the protocol defined by Staudt et al. [69]. During the TMS assessment, the children will wear a cap that allows to create a coordinate system used to find the optimal point to stimulate (hotspot) in a systematic way for all participants. The hotspot and the resting motor threshold (RMT) are identified by starting the stimulation intensity at 30% and increasing it in steps of 5%. The RMT is defined as the minimum intensity needed to obtain 5 out of 10 MEP of at least 50 μV in the correspondent muscle. After hotspot and RMT identification, 10 MEPs will be collected at an intensity of 120% the RMT. The TMS session is carried out as follows: first, stimulation starts in the less-affected hemisphere, where contralateral projections to the contralateral hand are searched and identified. Second, stimulation in the less-affected hemisphere continues up to 100% of the maximum stimulator output to search for possible ipsilateral projections to the ipsilateral (more impaired) hand. Third, we stimulate the affected hemisphere to search for possible contralateral projections to the contralateral hand (more impaired hand). If only contralateral MEPs from each hemisphere are found, the child will be categorized as having a contralateral CST wiring pattern. If MEPs in the more impaired hand are identified from both hemispheres, the child will be categorized as having a bilateral CST wiring pattern. Lastly, if MEPs in the impaired hand are only found when stimulating the less-affected hemisphere (ipsilateral hemisphere), the child will be categorized as having an ipsilateral CST wiring.

### Data management

To assure anonymity, a study-specific participant-identifier will be assigned to each participant upon enrollment. A participant identification code list will be generated, including contact details, and will be stored separately. Descriptive data (clinical assessments including videos, digital questionnaire responses, activity logs) and other raw and/or processed data (brain imaging and neurophysiology data, kinematics) will be collected and stored as software-specific data files on a secured network using the anonymous study-specific participant-identifier.

LM and CSM will be the investigators with access to the personal data and will be responsible for its anonymization as well as for ensuring data quality (double data entering, data values range checks, outliers detection). The final trial dataset will be accessible by LM, CSM, KK and HF.

### Statistical analysis

Descriptive statistics of the outcome variables will be reported by using means and standard deviations or median and interquartile ranges, depending on their data distribution. Normality will be checked with the Shapiro-Wilk test and histograms will be checked for symmetry. Mixed models will be used to study changes after the intervention over time. By using random effects, these models are able to correct for the dependency among repeated observations. Furthermore, these models deal with missing data offering valid inferences, assuming that missing observations are unrelated to unobserved outcomes [70]. Based on the data distribution, linear (parametric) or generalized (non-parametric) linear mixed models will be used. Changes over time will be tested between groups, by analysing treatment-time interactions. In case of such a significant treatment-time interaction, changes over time will be investigated separately in each group. Significant time trends will be further investigated with pairwise post hoc tests to compare time points. Additionally, the effect size will be calculated using the Cohen’s d formula (small, 0.2–0.5; medium, 0.5–0.8, and large > 0.8) [71]. Both clinical (age, baseline AHA score, sensory function, mirror movements) and neurological predictors (brain lesion characteristics, structural and functional connectivity and CST wiring) will be included as covariates in the models for the primary outcome measure, together with their interaction with time and treatment to evaluate their potential confounding factor. The two-sided 5% level of significance will be used. All statistical analyses will be performed using SAS version 9.2 (SAS Institute, Inc., Cary, NC) and SPSS Statistics for Windows version 24.0 (IBM Corp. Armonk, NY: IBM Corp.).

## Discussion

This paper presents the background and design for a single-blinded RCT comparing mCIMT in combination with AOT to mCIMT alone in children with uCP and investigating the role of different neurological biomarkers in predicting treatment response. To the best of our knowledge, this is the first study to investigate the added value of a novel treatment approach based on a neurophysiological model (AOT) to a motor execution treatment model (mCIMT). The outcomes across all domains of the ICF will be evaluated using valid and reliable clinical tools as well as 3DMA. Furthermore, the predictive value of neurological factors on treatment response will be investigated. This may be useful in predicting which children respond best to these training approaches and thus assist in an effective allocation of resources.

The results of this study will be disseminated through peer-reviewed publications as well as active participations at international conferences. Participating in activities and events aimed at the translation of science will bring our research results to a broader audience (local clinicians, parents, and children).

## Additional files

Additional file 1: **Table S1.** Description of the goal-directed actions of during AOT for children with a House Functional Classification 4–5. (DOCX 19 kb)

Additional file 2: **Table S2.** Description of the goal-directed actions of during AOT for children with a House Functional Classification 6–8. (DOCX 18 kb)

## Abbreviations

3DFLAIR: Three dimensional fluid-attenuated inversion recovery
3DMA: Three dimensional movement analysis
AHA: Assisting Hand Assessment
AOT: Action-Observation Training
CC: Corpus callosum
CP: Cerebral Palsy
CST: Corticospinal tract
dMRI: Diffusion Magnetic Resonance Imaging
EMG: Electromyography
FOV: Field of view
HFC: House Functional Classification
HTH: Hand to head
HTM: Hand to mouth
HTS: Hand to shoulder
ICF: International Classification of Functioning, Disability and Health
JTHFT: Jebsen-Taylor hand function test
MA2: Melbourne Assessment 2
MACS: Manual Ability Classification system
mCIMT: Modified Constraint Induced Movement Therapy
MEPs: Motor-evoked potentials
MPRAGE: Magnetization prepared rapid gradient echo
MRI: Magnetic Resonance Imaging
pROM: Passive range of motion
RCT: Randomized controlled trial
RF: Reaching forward
RF: Reaching sideways
RGS: Reaching to grasp a sphere
RGV: Reaching to grasp a vertically oriented cylinder
RMT: Resting motor threshold
RS: Reaching sideways
rsfMRI: Resting state functional Magnetic Resonance Imaging
SPM: Statistical Parametric Mapping
TE: Echo time
TMS: Transcranial magnetic stimulation
TR: Repetition time
uCP: Unilateral cerebral palsy
UL: Upper limb
