# Supplementary material for: Combining constraint-induced movement therapy and action-observation training in children with unilateral cerebral palsy: a randomized controlled trial
Source: BMC Pediatr. 2018 Jul 31;18:250. doi: 10.1186/s12887-018-1228-2 (PMC6069849; doi:10.1186/s12887-018-1228-2)
Supplement: Supplementary file 2 — Table S2. Description of the goal-directed actions of during AOT for children with a House Functional Classification 6–8. (DOCX 18 kb) [file 12887_2018_1228_MOESM2_ESM.docx]

**Combining constraint-induced movement therapy and action-observation training in children with unilateral cerebral palsy: a randomized controlled trial**

Cristina Simon-Martinez^1¥^*, Lisa Mailleux^1¥^, Els Ortibus^2^, Anna Fehrenbach^1^, Giuseppina Sgandurra^3,4^, Giovanni Cioni^3,4^, Kaat Desloovere^1,5^, Nicole Wenderoth^6^, Philippe Demaerel^7^, Stefan Sunaert^7^, Guy Molenaers^2^, Hilde Feys^1§^ and Katrijn Klingels^1,8 §^

^1^ KU Leuven - University of Leuven, Department of Rehabilitation Sciences, Leuven, Belgium

^2^ KU Leuven - University of Leuven, Department of Development and Regeneration, Leuven, Belgium

^3^ Department of Developmental Neuroscience, IRCCS Fondazione Stella Maris, Calambrone, Italy

^4^ Department of Clinical and Experimental Medicine, University of Pisa, Pisa, Italy

^5^ University Hospitals Leuven, Clinical Motion Analysis Laboratory, Pellenberg, Belgium

^6^ Neural Control of Movement Lab, Department of Health Sciences and Technology, ETH Zurich, Switzerland

^7^ University Hospitals Leuven, Department of Radiology, Leuven, Belgium

^8^ Rehabilitation Research Centre, BIOMED, Hasselt University, Diepenbeek, Belgium

¥ These authors have contributed equally to this work

§ These authors have contributed equally to this work

* Corresponding author:

Cristina Simon-Martinez

cristina.simon@kuleuven.be

Table S2. Description of the goal-directed actions of during AOT for children with a House Functional Classification 6-8.

| **Activity** | **Description of sub-activity 1** | **Description of sub-activity 2** | **Description of sub-activity 3** |
| --- | --- | --- | --- |
| 1 | Lift the box covering a candy and place it next to the candy | Pick up a candy from the table and drop it in a cup | Pour water from a small plastic bottle into a cup containing a candy and put the bottle back |
| 2 | Pick up a card from a small wooden block and place it on an identical card on a raised rectangular box | Pick up a colored card from a card holder and place it on a square with the same color | Pick up a colored card (located on a raised rectangular box), turn it over, and lay it down |
| 3 | Pick up an animal-shaped sponge stamp and place it in a circle on a paper | Pick up an animal-shaped sponge stamp and make a print in a circle on a paper, and put the stamp back (stamp at the middle of the upper side of the paper) | Pick up an animal-shaped sponge stamp and make a print in a circle on a vertical paper, and put the stamp back |
| 4 | Pick up a coin from a holder and put it into a box through a vertical slot | Pick up a coin from a holder and put it into a box through a horizontal slot | Pick up a coin from a holder and put it into a box through an oblique slot |
| 5 | Pick up a small stamp and place it on a drawn circle on a paper | Pick up a small stamp on the right, make a print in a drawn circle on a paper, and put it back | Pick up a small stamp on the top of the paper, make a print in a circle on a paper, and put it back |
| 6 | Pick up a spray can and place it on a paper more to the left | Pick up a spray can and lay it down in a shaped foam | Spray with the spray can (fixed in a Styrofoam shell) into a cup (positioned on the shaped foam) |
| 7 | Pick up a tube with a cap containing glitter powder and place it down to the left or right | Lift the cap from a tube containing glitter powder and put it on the table | Pick up the tube without a cap, and pour some glitter powder on a drawing, and place it back |
| 8 | Pick up a magnet and place it on a piece of paper | Pick up a toy fishing rod, catch the animal magnet with it and put the rod in a large cup | Take the animal magnet from the fishing rod in the cup and put it in a small plate |
| 9 | Grab a cloth that is spread out, and put it into a bowl filled with water | Pick up a bottle of detergent, pour some into the bowl and place it back | Pick up the cloth from the water in the bowl, wring it out and put it in a bucket on the right |
| 10 | Take a wooden clip from an edge of a box on a higher level and lay it down (wooden clip on his side) | Pick up a clip from a small wooden block and put it horizontally on the tale of a cardboard animal | Pick up a clip from a small wooden block and put it vertically on the tale of a cardboard animal |
| 11 | Place your hand on a ball of clay and flatten it | Move a clay form from a paper to the middle of the flattened clay | Make a pattern in the clay by pressing on the top of the form, pick up the form and put it on a paper |

Table S2 (continuation).

| **Activity** | **Description of sub-activity 1** | **Description of sub-activity 2** | **Description of sub-activity 3** |
| --- | --- | --- | --- |
| 12 | Pick up a tube of paint, squeeze some of the paint in a plastic bowl and put the tube back | Pick up a brush and put it in the bowl filled with some paint | Pick up the brush in the bowl, color the paper using a template, and put the brush back in the bowl |
| 13 | Pick up a seed from the table and drop it in a flower pot | Pick up a small sand shovel from a bin filled with sand, pour it in the pot and place the shovel back in the bin | Lift the watering can, pour some water in the pot filled with sand, and place it back |
| 14 | Pick up a spider from a web that is placed on the table, and drop it in a can on the right | Lift a cut-out part of a box with a handgrip, and place it next to the box | Lift the can filled with the spiders, pour it out in the box, and place the can back on the table |
| 15 | Pick up a plastic bag filled with short pieces of colored wire, and put the bag in a large bowl | Take the plastic bag out of the bowl, so that the pieces of wire remain in the bowl, and place the bag next to the bowl | Pick up a clamp next to the bowl, grab some pieces of wire in the bowl, put them on a drawing next to the bowl and place the clamp back on the table |
